# Mesoporous Strontium-Doped Phosphate-Based Sol-Gel Glasses for Biomedical Applications

**DOI:** 10.3389/fchem.2020.00249

**Published:** 2020-04-23

**Authors:** Farzad Foroutan, Benjamin Alexander Kyffin, Isaac Abrahams, Jonathan C. Knowles, Elisa Sogne, Andrea Falqui, Daniela Carta

**Affiliations:** ^1^Department of Chemistry, University of Surrey, Guildford, United Kingdom; ^2^School of Biological and Chemical Sciences, Materials Research Institute, Queen Mary University of London, London, United Kingdom; ^3^Division of Biomaterials and Tissue Engineering, University College London, Eastman Dental Institute, London, United Kingdom; ^4^The Discoveries Centre for Regenerative and Precision Medicine, London, United Kingdom; ^5^Department of Nanobiomedical Science & BK21 PLUS NBM Global Research Centre for Regenerative Medicine, Dankook University, Cheonan-si, South Korea; ^6^UCL Eastman-Korea Dental Medicine Innovation Centre, Dankook University, Cheonan-si, South Korea; ^7^NABLA Lab, Biological and Environmental Sciences and Engineering (BESE) Division, King Abdullah University of Science and Technology (KAUST), Thuwal, Saudi Arabia

**Keywords:** phosphate-based glasses, strontium-doped, tissue engineering, bioresorbable glasses, sol-gel

## Abstract

Mesoporous phosphate-based glasses have great potential as biomedical materials being able to simultaneously induce tissue regeneration and controlled release of therapeutic molecules. In the present study, a series of mesoporous phosphate-based glasses in the P_2_O_5_-CaO-Na_2_O system, doped with 1, 3, and 5 mol% of Sr^2+^, were prepared using the sol-gel method combined with supramolecular templating. A sample without strontium addition was prepared for comparison. The non-ionic triblock copolymer EO_20_PO_70_EO_20_ (P123) was used as a templating agent. Scanning electron microscopy (SEM) images revealed that all synthesized glasses have an extended porous structure. This was confirmed by N_2_ adsorption-desorption analysis at 77 K that shows a porosity typical of mesoporous materials. ^31^P magic angle spinning nuclear magnetic resonance (^31^P MAS-NMR) and Fourier transform infrared (FTIR) spectroscopies have shown that the glasses are mainly formed by Q^1^ and Q^2^ phosphate groups. Degradation of the glasses in deionized water assessed over a 7-day period shows that phosphate, Ca^2+^, Na^+^, and Sr^2+^ ions can be released in a controlled manner over time. In particular, a direct correlation between strontium content and degradation rate was observed. This study shows that Sr-doped mesoporous phosphate-based glasses have great potential in bone tissue regeneration as materials for controlled delivery of therapeutic ions.

## Introduction

In recent years, mesoporous glasses have gained increased attention in the field of functional biomaterials (Baino et al., 2016). Their extended porosity, high surface areas and high pore volumes facilitate the interaction between the biomaterial and biological fluids. Moreover, therapeutic ions and molecules can be incorporated in the mesopores (pores size range 2–50 nm) with high loadings and released in a controlled manner (Wu and Chang, 2014). A significant amount of work has been performed on the synthesis of mesoporous silicate-based glasses mainly as drug delivery systems (Vallet-Regí et al., 2007; Yang et al., 2012) and for bone tissue regeneration applications (Zhang et al., 2016). However, only one very recent work has been presented on undoped mesoporous phosphate-based glasses (Foroutan et al., 2020), for which the synthesis has been considered to be “*a significantly challenging area for future efforts*” in a recent review on sol-gel based materials for biomedical applications (Owens et al., 2016). In contrast to silicate-based glasses, phosphate-based glasses (PGs) have the particular property of being bioresorbable (Abou Neel et al., 2011). When in contact with body fluids, they slowly dissolve in the physiological environment and they are eventually totally replaced by regenerated tissue. Thanks to their complete solubility, PGs can be used as safe degradable temporary implants, avoiding the necessity of a second operation for their surgical removal (Knowles, 2003). As ions released from these glasses already exist in the body, problems related to toxicity and inflammatory reactions are avoided (Foroutan et al., 2015). However, silicate-based glasses have a poor solubility and typically they take several years to dissolve in a physiological environment (Fragogeorgi et al., 2019). Therefore, they cannot be used as temporary implants and their long-term effects in the body are still unknown. In addition, PGs offer advantages over polymer-based bioresorbable systems, as they do not leave any crystalline products upon dissolution. Release of crystalline fragments with heterogeneous chain-lengths during degradation of bioresorbable polymers has been reported to cause inflammatory reactions (Cheung et al., 2007; Sabir et al., 2009). Given their bioresorbability, PGs can be used as controlled local delivery systems of therapeutic species (*e.g*., antimicrobial ions/growth factors) that can be released in a controlled manner as the implant degrades. Controlled delivery avoids the need for oral administration and injection, improving the quality of life of patients (Wanakule and Roy, 2012; Wilczewska et al., 2012). The introduction of mesoporosity into PGs is expected to enhance the potential applications of these materials as controlled drug delivery systems, as the majority of ions/drugs used in clinical practice can easily be hosted in the mesopores (Vallet-Regí et al., 2007; Martínez-Carmona et al., 2018).

The conventional method to prepare PGs is the high temperature melt quenching technique (MQ), which involves heating the oxide precursors to temperatures in excess of 1,000°C (Ahmed et al., 2006; Al Qaysi et al., 2015). However, this method cannot be used for the production of porous PGs. Moreover, MQ often leads to non-homogeneous, bulk glasses that cannot be used for hosting temperature sensitive molecules. Recent studies have shown that PGs can be synthesized via the sol-gel method (SG) at much lower processing temperatures (Carta et al., 2005, 2007; Pickup et al., 2007; Foroutan et al., 2014). The sol-gel method is a wet chemical bottom-up technique based on the hydrolysis and polycondensation of alkoxide precursors in solution (Pickup et al., 2007; Owens et al., 2016). The use of precursors in solution allows the production of highly homogeneous glasses with controlled morphology (monoliths, porous foams, fibers, spheres, and thin films) (Carta et al., 2007; Lee et al., 2013). Additionally, by using the SG method, surfactant molecules can be easily added into the precursor solutions. Therefore, the SG process is ideal for the synthesis of mesoporous systems.

The surfactants spontaneously self-assemble in specific-shaped micelles at the critical micellar concentration, the shape and size depending on the specific surfactant used (Izquierdo-barba and Vallet-Regí, 2015). After removal of the surfactant via calcination, pores having the sizes of the micelles are left in the inorganic material. The morphology of pores can be tailored thanks to the easily controlled solution-based chemistry.

In this study, mesoporous glasses in the P_2_O_5_-CaO-Na_2_O system doped with 1, 3, and 5 mol% of Sr^2+^ were successfully synthesized by the sol-gel method using the non-ionic block copolymer P123 as a templating agent. PGs in the P_2_O_5_-CaO-Na_2_O system have been shown to be effective materials for osteogenesis and controlled release of Ca^2+^ and Na^+^ ions as passive host materials for veterinary treatments (Abou Neel et al., 2011). There is great interest in doping these systems with functional ions. Several studies have been reported on P_2_O_5_-CaO-Na_2_O glasses doped with antibacterial ions such as Cu^2+^, Ag^+^, Zn^2+^, and Ga^3+^ (Valappil et al., 2008; Abou Neel et al., 2011; Qaysi et al., 2016; Foroutan et al., 2019; Kyffin et al., 2019). During the last decade, the interest in the addition of Sr^2+^ to materials for bone regeneration has greatly increased due to the development of strontium ranelate as a promising drug for treating osteoporosis. Strontium has a unique effect on bone and can simultaneously promotes bone formation and inhibits bone resorption (Querido et al., 2016; Sriranganathan et al., 2016). Incorporation of strontium into silicate-based glasses has been shown to increase cell proliferation and normalized alkaline phosphatase activity in human osteosarcoma bone cells. Sr^2+^ also inhibited calcium phosphate resorption and reduced tartrate-resistant acid phosphatase activity, suggesting a dual action mechanism of released Sr^2+^ ions (Gentleman et al., 2010). Recent *in vivo* studies also demonstrated the effectiveness Sr-doped silicate-based glasses in promoting bone cells differentiation and proliferation in rabbits (Newman et al., 2014). In PGs, strontium has been incorporated only into MQ prepared systems (Abou Neel et al., 2009a; Al Qaysi et al., 2015; Sriranganathan et al., 2016). These studies confirmed the potential applications of Sr-doped PGs as effective vehicles to deliver Sr^2+^ to bone cells (Abou Neel et al., 2009a; Lakhkar et al., 2011). These glasses can also promote osteogenic differentiation of human mesenchymal stem cells that make them a great choice in various orthopedic, dental, and maxillofacial applications (Stefanic et al., 2018).

The majority of the P_2_O_5_-CaO-Na_2_O systems presented in the literature have been prepared via MQ; much less work has been done on analogous non-porous systems prepared via SG. In particular, no previous examples of mesoporous strontium-doped phosphate-based glasses have been presented in the literature. Therefore, here we presented for the first time a study on the sol-gel synthesis and characterization of strontium doped mesoporous phosphate-based glasses and the effect of strontium addition on dissolution properties.

## Materials and Methods

### Synthesis

The following chemical precursors were used without further purification; n-butyl phosphate (1:1 molar ratio of mono OP(OH)_2_(OBu^n^) and di-butyl phosphate OP(OH)(OBu^n^)_2_, Alfa Aesar, 98%), calcium methoxyethoxide (Ca-methoxyethoxide, ABCR, 20% in methoxyethanol), sodium methoxide solution (NaOMe, Aldrich, 30 wt% in methanol), strontium acetate (Sr-acetate, Aldrich, 97%), ethanol (EtOH, Fisher, 99%), and Pluronic (P123-M_n_ = 5,800, Aldrich).

**Figure 1:**
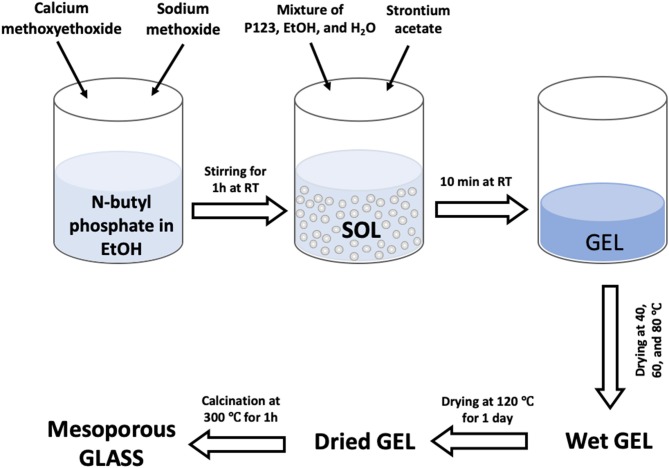
Flow diagram for the sol-gel synthesis of Sr-doped MPGs.

The target compositions in mol% of glasses were (P_2_O_5_)_0.55_-(CaO)_0.30_-(Na_2_O)_0.15−x_-(SrO)_x_, with x = 1, 3, or 5. In order to prepare the undoped glass, 1.7 g of n-butyl phosphate was added to 5 mL of EtOH in a dried vessel and left under stirring for 10 min. 3.5 g of Ca-methoxyethoxide and 0.5 g of NaOMe were then added dropwise into the mixture while stirring; the solution was kept under stirring for about 1 h. In order to prepare the Sr-doped glasses 0.04, 0.11, or 0.18 g of Sr-acetate were added to the mixture to prepare glasses containing x = 1, 3, or 5 mol% SrO, respectively; the quantity of NaOMe added was reduced accordingly. The mixtures were allowed to react for a further 10 min. Finally, a solution consisting of 3.0 g P123, 5 mL EtOH and 2.5 mL H_2_O was added to the mixtures and allowed to react for 10 min. The mixtures were poured into glass containers and allowed to gel at room temperature. Gelation occurred after about 5 min; gels were then aged for 1 day at room temperature. Then the temperature was increased from room temperature to 40°C and held for 1 day, to 60°C and held for 2 days, then to 80°C and held for 2 days, and finally to 120°C and held for 1 day before calcination at 300°C at a heating rate of 1°C·min^−1^ to remove surfactant and solvents from the samples. The obtained glasses were ground at 10 Hz to form microparticles (MM301 milling machine, Retsch GmbH, Hope, UK); microparticles in the size range of 106–200 μm were obtained using test sieves (Endecotts Ltd, London, UK). The sol-gel preparation of Sr-doped glasses is outlined in the flowchart in Figure 1. Glasses will be hereafter indicated MPG-und (undoped) and MPG-SrX (doped) where X = mol% SrO.

### Structural Characterization

X-ray powder diffraction (XRD, PANalytical X'Pert, Royston, UK) was performed on powdered samples in flat plate θ/θ geometry using Ni filtered Cu Kα radiation. Data were collected using a PIXcel^1D^ detector with a step size of 0.0525° over a 2θ range of 10–90° and a time per step of 12 s.

SEM images reported in the main manuscript were performed with a JSM-7100F instrument (Jeol, Welwyn, UK) at an accelerating voltage of 10.0 kV and working distance of 10 mm. The samples were mounted onto aluminum stubs using carbon conductive tape. Pore sizes were measured using the Image-pro plus software (Media Cybernetics, USA). In order to determine the exact compositions of the prepared samples and compare them with the theoretical compositions energy dispersive X-ray spectroscopy (EDX, MagnaRay, ThermoFisher, Hemel Hempstead, UK) was performed using an SEM operating at 20.0 kV.

SEM images reported in the Supplementary Information were obtained by High-Resolution SEM (HRSEM) at very low acceleration voltage, using a FEI Magellan microscope equipped with a Field Emission Gun (FEG) electron source, imaging at high magnification the uncoated materials with an acceleration voltage of 1 kV, a probe current of 6.3 pA, an in-lens secondary electron detector and a monochromator (the FEI Wien filter called UC), the latter allowing for work with an energy spread of 0.15 eV FWHM, in order minimize any sample change induced by the electron beam, concomitantly getting a very surfacial imaging with an ultimate lateral resolution of <1 nm.

Solid state ^31^P MAS-NMR spectra (AVANCE III, Bruker, Coventry, UK) were recorded at 161.87 MHz using direct excitation with a 90° pulse and 60 s recycle delay at ambient probe temperature (~25°C). Between 20 and 88 scans were acquired in each case. Powder samples were loaded into 4.0 mm outer diameter zirconia rotors and spun at 12 kHz. Spectra were referenced to the resonance of the secondary reference ammonium dihydrogen phosphate (NH_4_H_2_PO_4_) at 0.9 ppm (relative to 85% H_3_PO_4_ solution at 0 ppm). Spectra were fitted using the Dmfit software package (Massiot et al., 2002).

FTIR spectra for each sample were acquired using a FTIR-2000 instrument equipped with Timebase software (Perkin Elmer, Seer Green, UK) with an attenuated total reflectance accessory (Golden Gate, Specac, Orpington, UK). Samples were scanned at room temperature in absorbance mode in the range of 600–1,500 cm^−1^.

N_2_ adsorption-desorption analysis was performed on a Gemini V, Micromeritics, Hertfordshire, UK; in particular, the specific surface area was assessed by using the Brunauer-Emmet-Teller (BET) method, and the pores size distribution was determined from the desorption branch of the isotherm through the Broekhoff-de Boer (BdB) method and the Barrett-Joyner-Halenda (BJH) method.

### Degradation Study

10 mg of powdered samples were immersed in 10 mL deionized water for 1, 3, 5, and 7 days (*n* = 3) at 37 °C. The resulting suspensions for each time point were then centrifuged at 4,800 rpm for 10 min to separate the powder glasses from the solution. Phosphorus, calcium, sodium, and strontium in solution were subsequently measured by ICP-OES (720ES-Varian, Crawley, UK) calibrated across the predicted concentration range using standard premade solutions (ICP multi-element standard solution, VWR). Both samples and standards were diluted in a 1:1 ratio with 4% HNO_3_ (Honeywell, Fluka™). A blank (2% HNO_3_) solution was used as a reference under standard operating conditions. Simulated body fluid (SBF) or phosphate buffered saline (PBS) as alternative media solutions were not used as they contain Ca^2+^, Na^+^ and phosphate anions that would have interfered with the quantification of the small amount of the same ions released from the glasses.

## Results and Discussion

**Figure 2:**
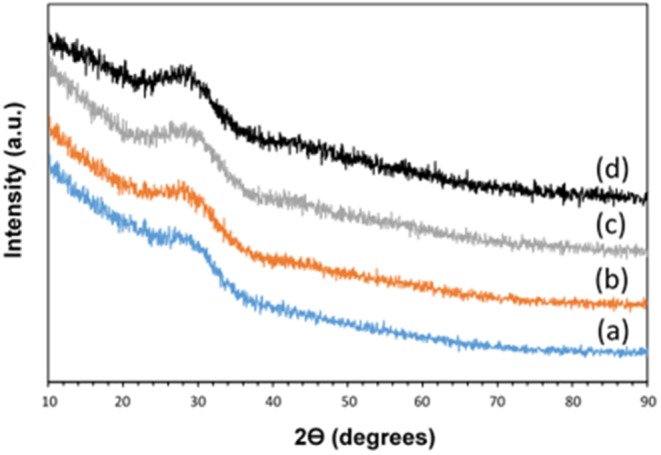
XRD patterns of (a) MPG-und, (b) MPG-Sr1, (c) MPG-Sr3, and (d) MPG-Sr5.

XRD was performed in order to assess glass formation and absence of crystallinity. The XRD patterns of all samples are reported in Figure 2. No Bragg peaks are present confirming the amorphous nature of the samples prepared. Only a broad halo centered at 2θ ~ 29° can be observed, due to the phosphate glass network.

The composition of the glasses was determined via chemical analysis carried out using SEM equipped with an EDX detector. Sample compositions in terms of mol% are reported in Table 1; elemental compositions in terms of weight % along with a representative EDX spectra are reported in Table S1 and Figure S1, respectively.

**Table 1:** Compositions of MPGs measured by EDX (mol%).

**Sample code**	**P_**2**_O_**5**_**	**CaO**	**Na_**2**_O**	**SrO**
MPG-und	45.0 ± 1.2	36.0 ± 1.0	19.0 ± 0.6	–
MPG-Sr1	45.3 ± 1.4	35.7 ± 0.9	17.9 ± 0.5	1.1 ± 0.2
MPG-Sr3	44.6 ± 0.8	36.4 ± 1.1	15.8 ± 0.9	3.2 ± 0.5
MPG-Sr5	45.1 ± 0.7	36.1 ± 0.6	13.7 ± 0.5	5.3 ± 0.3

The P_2_O_5_ content showed a reduction of around 9–10 mol% from the theoretical values. Previous studies on MQ and SG phosphate-based glasses have shown that a P_2_O_5_ content in the range 40–55 mol% and CaO content in the range 20–40 mol% have good bioactivity and biocompatibility (Carta et al., 2005; Abou Neel et al., 2009b; Al Qaysi et al., 2015).

**Figure 3:**
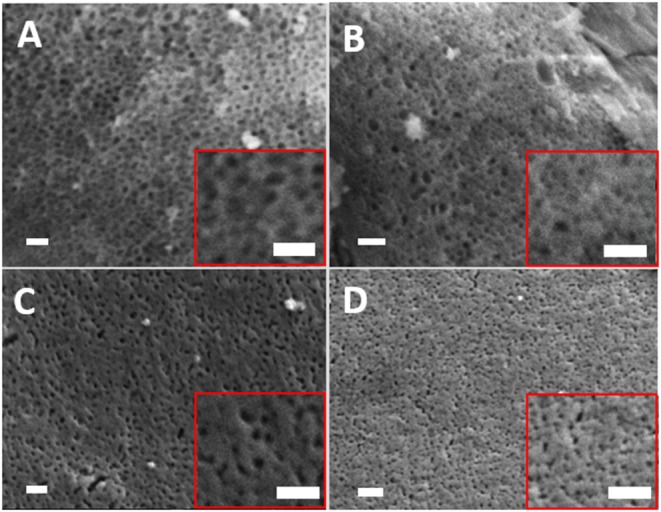
SEM images of **(A)** MPG-und, **(B)** MPG-Sr1, **(C)** MPG-Sr3, and **(D)** MPG-Sr5. In all images and insets scale bar is 200 nm.

The removal of the organic micelles of Pluronic (P123) by slow heating resulted in the formation of highly porous glasses. This is clearly shown in the SEM images reported in Figure 3. The mean pore sizes calculated from SEM images are 22, 21, 20, and 22 nm for MPG-und MPG-Sr1, MPG-Sr3, and MPG-Sr5, respectively. Histograms of pore size distribution obtained by statistical analysis of SEM images are reported in Figure S3.

**Figure 4:**
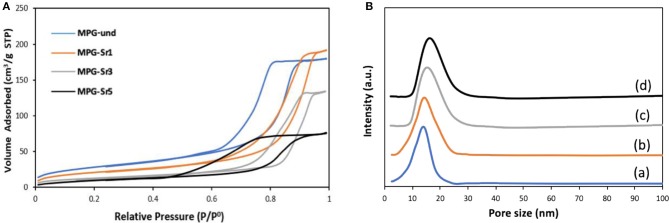
**(A)** N_2_ adsorption and desorption isotherms and **(B)** pore size distribution of MPG-und (a), MPG-Sr1 (b), MPG-Sr3 (c), and MPG-Sr5 (d).

Analysis of pore sizes was also performed by using N_2_ adsorption-desorption analysis at 77 K. The N_2_ adsorption-desorption isotherms and the pore size distribution curves for all the glasses are reported in Figures 4A,B, respectively. Surface areas and pore sizes are reported in Table 2.

**Table 2:** Textural properties of MPGs.

**Sample code**	**Surface area (m^2^·g^−1^)**	**Pore size (nm)**
MPG-und	123	11.8
MPG-Sr1	112	13.2
MPG- Sr3	94	15.1
MPG- Sr5	73	18.6

The N_2_ adsorption-desorption isotherms can be classified as type IV, characteristic of mesoporous solids. As the Sr^2+^ content increases, the shape of the isotherm changes in particular for the glass with the highest strontium content. The surface area decreases with increasing Sr^2+^ content from 123 m^2^·g^−1^ (MPG-und) to 73 m^2^·g^−1^ (MPG-Sr5). Even if the surface areas are lower than typical mesoporous silicate-based glasses, this is a remarkable result giving that the calcium phosphate-based glasses are known to have a much weaker network structure than that of silicate-based systems. Pore size does not change significantly with strontium content and pore size distributions are all quite narrow. However, a slight increase in pore size is observed from 11.8 nm (MPG-und) to 18.6 nm (MPG-Sr5). It has been previously observed that higher dopant loading leads to higher pore sizes and lower surface areas in sol-gel zinc-doped silicate-based glasses (Courthéoux et al., 2008). This was ascribed to the modification of the network caused by the addition of the dopant.

**Figure 5:**
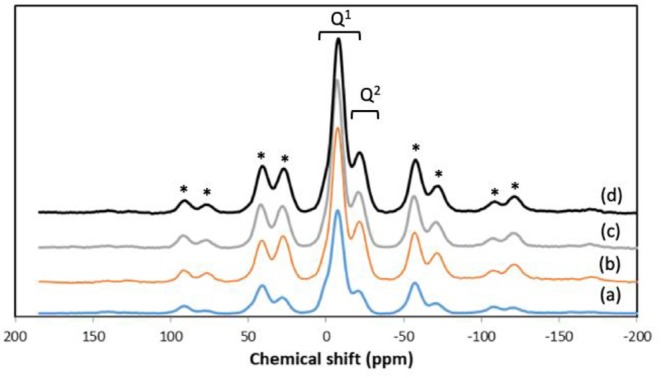
^31^P MAS-NMR spectra of (a) MPG-und, (b) MPG-Sr1, (c) MPG-Sr3, and (d) MPG-Sr5. Spinning sidebands marked with asterisks.

In addition to textural properties, the structure of the phosphate network can also play a very important role, in particular in relation to dissolution properties. Therefore, we have investigated the structure of the phosphate network using ^31^P MAS-NMR. This technique is a very powerful tool for the investigation of the connectivity of the phosphate units and the local environment around phosphorus. The ^31^P MAS-NMR spectra of all samples are presented in Figure 5 and the spectral parameters and assignments are reported in Table 3. Resonances are assigned to Q^*n*^ phosphate species, where *n* represents the number of bridging oxygens between phosphate units. All spectra look very similar, with resonances in the range −6.2/−6.8 ppm corresponding to Q^1^ groups and −22.9/−23.6 ppm corresponding to Q^2^ groups (Foroutan et al., 2019). However, as the Sr content increases, the intensity of Q^1^ groups decreases and the intensity of Q^2^ groups increases.

**Table 3:** ^31^P MAS-NMR spectral parameters (chemical shift, δ_iso_) and relative intensity (*I* %).

	**Q**^****1****^		**Q**^****2****^	

**Sample code**	***δ***_***iso***_ **(ppm)**	***I*** **% (±1)**	***δ***_***iso***_ **(ppm)**	*I* **% (±1)**
MPG-und	−6.2	68.3	−22.9	31.7
MPG-Sr1	−6.4	62.4	−23.0	37.6
MPG-Sr3	−6.5	61.9	−23.1	38.1
MPG-Sr5	−6.8	60.3	−23.6	39.7

**Figure 6:**
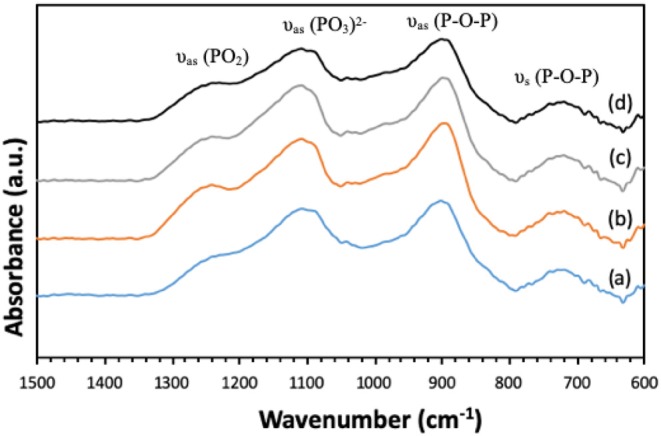
FTIR spectra of (a) MPG-und, (b) MPG-Sr1, (c) MPG-Sr3, and (d) MPG-Sr5.

Presence of Q^1^ and Q^2^ units in the phosphate chains was also confirmed by FTIR measurements. All FTIR spectra, reported in Figure 6, show similar bands, assigned on the basis of previous FTIR studies on phosphate-based glasses (Abou Neel et al., 2009a; Foroutan et al., 2019). The peak at 730 cm^−1^ can be assigned to the symmetrical stretching υ_s_ (P-O-P) mode, while the peak at 900 cm^−1^ can be assigned to the asymmetrical stretching υ_as_ (P-O-P) mode (Q^2^ phosphate units). The peaks at 1,100 and 1,235 cm^−1^ are also assigned to asymmetrical υ_as_ (PO_3_)^2−^ and υ_as_ (PO_2_) modes that can be related to Q^1^ and Q^2^ phosphate units, respectively.

**Figure 7:**
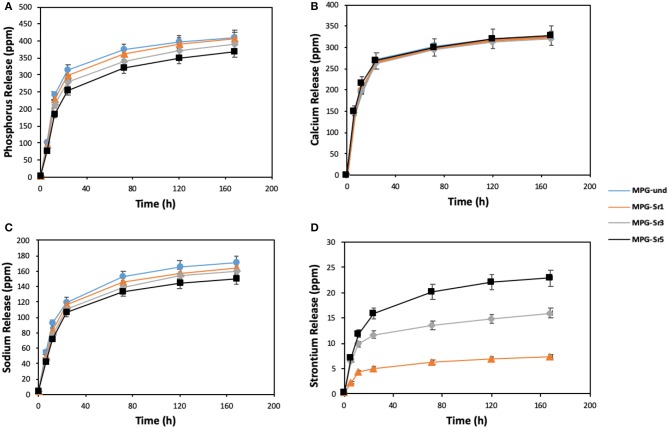
Release of **(A)** phosphorus, **(B)** calcium, **(C)** sodium, and **(D)** strontium in deionized water measured by ICP-OES. Error bars are SD (*n* = 3).

In addition to structural characterization, assessment of glass degradation, and quantification of ion delivery over time was performed. ICP-OES analysis was carried out to determine the release profiles of P, Ca, Na, and Sr following immersion of the glasses in deionized water up to 7 days (Figure 7).

The highest release of phosphate ions and Na^+^ is observed for the Sr^2+^-free glass (MPG-und). Phosphate ions and Na^+^ release decrease with increasing Sr^2+^ content, being the decrease more significant after the first 24 h.

These results suggest that the solubility of the glasses decreases by adding Sr^2+^; the addition of Sr^2+^ results in the formation of a more cross-linked glass structure, with increased stability of the glass network and hence, decrease the leaching of ions from glass network. This is in agreement with previous results on Sr^2+^ doped MQ glasses (Al Qaysi et al., 2015; Kargozar et al., 2019). Furthermore, the decrease in Na^+^ content with increasing Sr^2+^ amount can also be explained considering that Sr^2+^ was added in place of Na^+^ in the synthesis (Table 1). Therefore, as the Sr^2+^ content is increased, the amount of Na^+^ available to be released is decreasing.

Interestingly, calcium release profiles do not change significantly with strontium loading. This suggests that Ca^2+^ release is less affected by the decrease in glass solubility caused by Sr^2+^ addition. Similar results were obtained using strontium-doped phosphate-based glasses prepared via MQ (Stefanic et al., 2018). Ca^2+^ release was found to be independent of strontium loading; its release over time was very similar for systems containing 1, 5, and 10 mol% of SrO. Finally, Sr^2+^ release increases with SrO content as expected, as higher amounts of Sr^2+^ are available to be released in the glasses with higher Sr^2+^ content. Sr^2+^ ion release shows that is possible to control the amount of Sr^2+^ released over time. The morphology of the glasses after immersion was also investigated. SEM images of the representative sample MPG-Sr3 after 1, 3, 5, and 7 days of immersion in deionized water are shown in Figure S4. The images show that high porosity is preserved even after 7 days of immersion.

To conclude, this study has shown that strontium-doped phosphate-based glasses with an extensive mesoporosity and high surface area can be prepared using the sol-gel technique. Moreover, it has been demonstrated that Sr^2+^, which is known to play an important role in bone tissue regeneration, can be delivered in a controlled way over time.

Given that MPG-und has very recently been shown to have enhanced bioactivity and biocompatibility compared to analogous non-porous PGs (Foroutan et al., 2020), strontium doped MPG are expected to show even higher enhancement in bone cell viability. The promising findings encourages further studies to evaluate the potential of these glasses for bone tissue regeneration and drug delivery.

## Conclusions

Mesoporous phosphate-based glasses in the P_2_O_5_-CaO-Na_2_O system doped with Sr^2+^ = 1, 3, or 5 mol% were successfully prepared using the sol-gel method combined with the use of the surfactant block copolymer Pluronic 123. N_2_ adsorption-desorption analysis at 77 K of all glasses shows isotherms that can be classified as type IV, characteristic of mesoporous solids. The surface area of the undoped P_2_O_5_-CaO-Na_2_O glass is 123 m^2^·g^−1^; this value decreases upon addition of Sr^2+^ being 73 m^2^·g^−1^ for the sample doped with 5 mol% of Sr^2+^. ^31^P MAS-NMR and FTIR results revealed that the glass structure consists of mainly Q^1^ and Q^2^ phosphate units. Degradation of glasses in deionized water shows that as the Sr^2+^ content increases, the glass degradation rate decreases. The results show that the prepared mesoporous Sr-doped phosphate-based glasses have potential as materials for controlled delivery of therapeutic ions, in particular for bone repair applications.

## Data Availability Statement

The raw data supporting the conclusions of this article will be made available by the authors, without undue reservation.

## Author Contributions

FF performed synthesis and characterization of MPG and PG. BK has helped with characterization (IR and SEM). IA has provided access to MAS NMR and assisted with ^31^P MAS NMR data analysis. JK has provided access to ICP and assisted with dissolution data analysis. ES and AF have performed SEM imaging at high resolution. DC has lead the work and given expertise on data interpretation on all characterization techniques.

## Conflict of Interest

The authors declare that the research was conducted in the absence of any commercial or financial relationships that could be construed as a potential conflict of interest.
