# Supplementary material for: Mesoporous Strontium-Doped Phosphate-Based Sol-Gel Glasses for Biomedical Applications
Source: Front Chem. 2020 Apr 23;8:249. doi: 10.3389/fchem.2020.00249 (PMC7191082; doi:10.3389/fchem.2020.00249)
Supplement: Supplementary file 1 [file Data_Sheet_1.pdf]

Supplementary Information

Mesoporous strontium-doped phosphate-based sol-gel glasses for biomedical applications

F. Foroutan, [a] *B. A. Kyffin,* [a] *I. Abrahams,* [b] *J. C. Knowles,* [c, d, e, f] *, E. Sogne,* [g] *A. Falqui,* [g] *D. Carta* [a,*]

[a] *Department of Chemistry, University of Surrey, GU2 7XH, Guildford, UK.*

[b] *Materials Research Institute, School of Biological and Chemical Sciences, Queen Mary, University of London, Mile End Road, London E1 4NS, UK.*

[c] *Division of Biomaterials and Tissue Engineering, University College London, Eastman Dental Institute, 256 Gray's Inn Road, London WC1X 8LD, UK.*

[d] *The Discoveries Centre for Regenerative and Precision Medicine, UCL Campus, London, UK.*

[e] *Department of Nanobiomedical Science & BK21 PLUS NBM Global Research Centre for Regenerative Medicine, Dankook University, Cheonan 31114, Republic of Korea.*

[f] *UCL Eastman-Korea Dental Medicine Innovation Centre, Dankook University, Cheonan 31114, Republic of Korea.*

[g] *King Abdullah University of Science and Technology (KAUST), Biological and Environmental Sciences and Engineering (BESE) Division, NABLA Lab, 23955-6900 Thuwal, Saudi Arabia.*

Table S1: Compositions MPGs measured by EDX (weight %).

Sample code	Elemental composition (weight %)				
	P	Ca	Na	Sr	O
**MPG-und**	29.1±0.9	15.0±0.6	8.8±0.4	0.0±0.2	47.1±1.1
**MPG-Sr1**	28.9±0.7	15.4±0.5	8.7±0.5	2.5±0.4	44.5±0.9
**MPG-Sr3**	28.4±0.8	14.9±0.7	7.4±0.5	5.6±0.6	43.7±0.7
**MPG-Sr5**	28.5±1.0	14.7±0.4	6.3±0.4	8.6±0.5	41.9±1.0

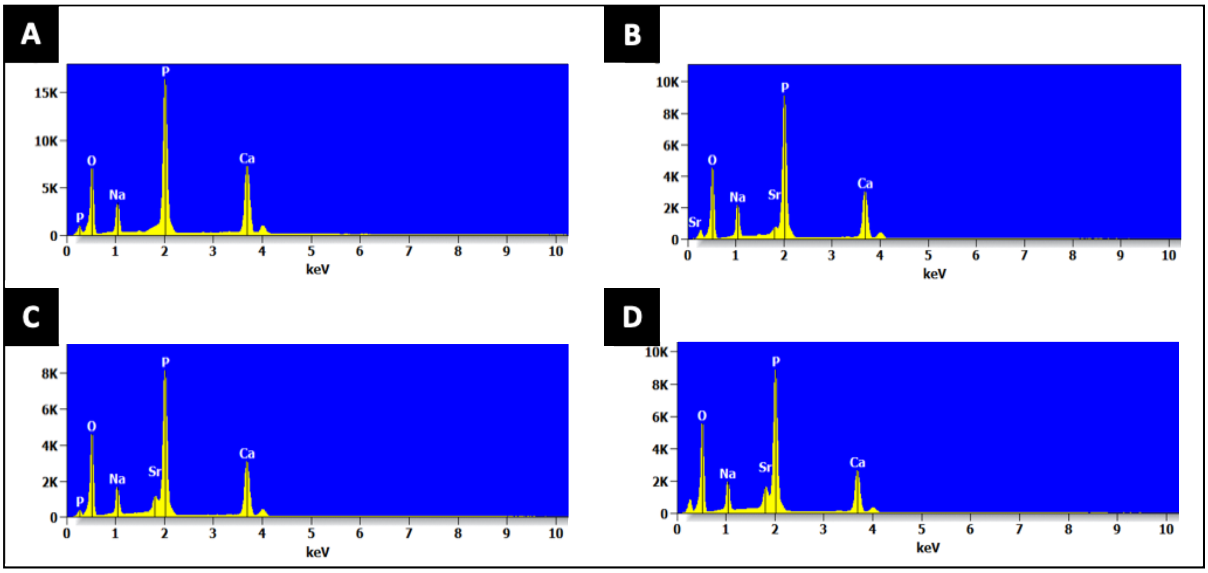

Figure S1. EDX spectra of **(A)** MPG-und, **(B)** MPG-Sr1, **(C)** MPG-Sr3 and **(D)** MPG-Sr5.

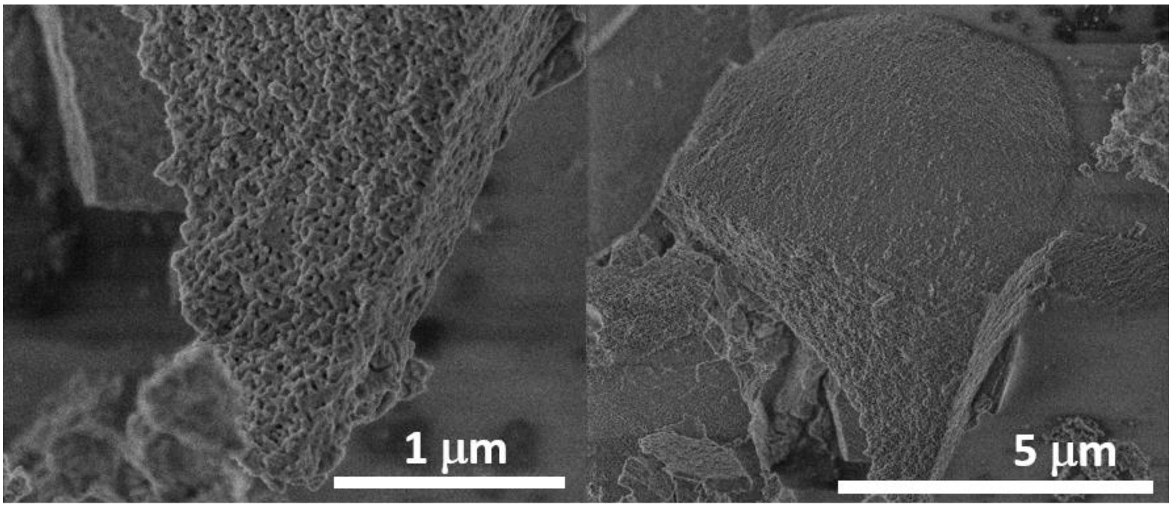

Figure S2. High-Resolution SEM at very low acceleration voltage of MPG-und.

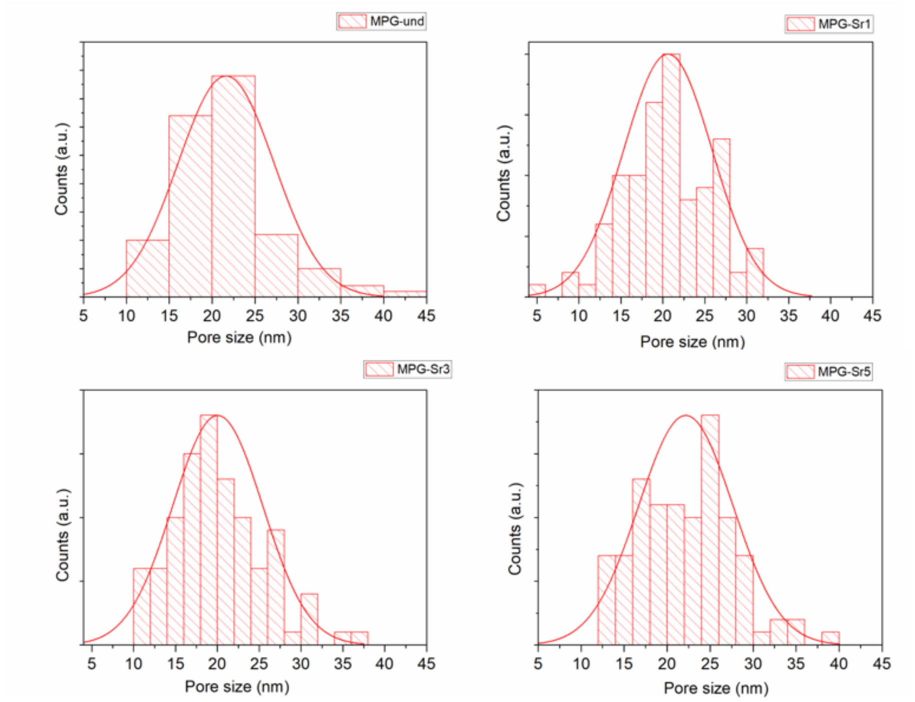

Figure S3. Histograms of pore size distribution obtained by statistical analysis of SEM images for MPG-und, MPG-Sr1, MPG-Sr3 and MPG-Sr5.

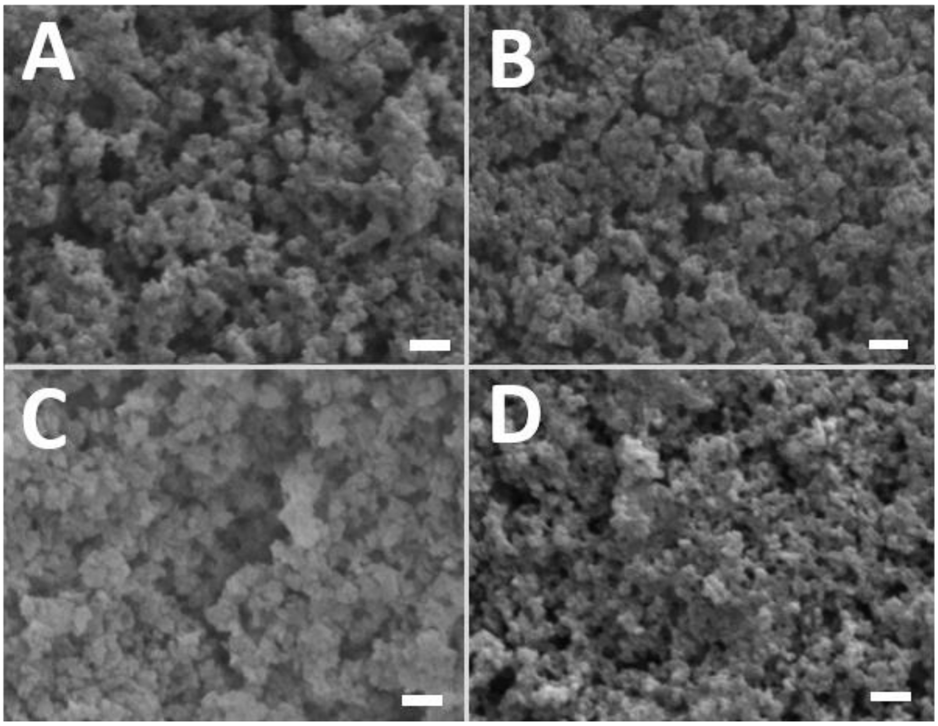

Figure S4. SEM images of MPG-Sr3 after immersion in deionized water for 1 day (A), 3 days (B), 5 days (C), 7 days (D). Scale bar = 200 nm.
